# Quantitative CT analysis of lung parenchyma to improve malignancy risk estimation in incidental pulmonary nodules

**DOI:** 10.1007/s00330-022-09334-w

**Published:** 2022-12-20

**Authors:** Alan A. Peters, Oliver Weinheimer, Oyunbileg von Stackelberg, Jonas Kroschke, Lars Piskorski, Manuel Debic, Kai Schlamp, Linn Welzel, Moritz Pohl, Andreas Christe, Lukas Ebner, Hans-Ulrich Kauczor, Claus Peter Heußel, Mark O. Wielpütz

**Affiliations:** 1 Diagnostic and Interventional Radiology, Heidelberg University Hospital, Im Neuenheimer Feld 420, 69120 Heidelberg, Germany; 2 Translational Lung Research Center Heidelberg (TLRC), German Center for Lung Research (DZL), Im Neuenheimer Feld 156, 69120 Heidelberg, Germany; 3 Department of Diagnostic and Interventional Radiology with Nuclear Medicine, Thoraxklinik at University of Heidelberg, Röntgenstraße 1, 69126 Heidelberg, Germany; 4 Department of Diagnostic, Interventional and Pediatric Radiology, Inselspital, Bern University Hospital, University of Bern, Freiburgstrasse, 3010 Bern, Switzerland; 5 Institute for Radiology, Kantonsspital Thurgau, Spitalcampus 1, 8596 Münsterlingen, Switzerland; 6 Institute of Medical Biometry, University of Heidelberg, Im Neuenheimer Feld 130.3, 69120 Heidelberg, Germany

**Keywords:** Lung neoplasms, Risk assessment, Decision support, Emphysema, Fibrosis

## Abstract

**Objectives:**

To assess the value of quantitative computed tomography (QCT) of the whole lung and nodule-bearing lobe regarding pulmonary nodule malignancy risk estimation.

**Methods:**

A total of 251 subjects (median [IQR] age*,* 65 (57–73) years; 37% females) with pulmonary nodules on non-enhanced thin-section CT were retrospectively included. Twenty percent of the nodules were malignant, the remainder benign either histologically or at least 1-year follow-up. CT scans were subjected to in-house software, computing parameters such as mean lung density (MLD) or peripheral emphysema index (pEI). QCT variable selection was performed using logistic regression; selected variables were integrated into the Mayo Clinic and the parsimonious Brock Model.

**Results:**

Whole-lung analysis revealed differences between benign vs. malignant nodule groups in several parameters, e.g. the MLD (−766 vs. −790 HU) or the pEI (40.1 vs. 44.7 %). The proposed QCT model had an area-under-the-curve (AUC) of 0.69 (95%-CI, 0.62−0.76) based on all available data. After integrating MLD and pEI into the Mayo Clinic and Brock Model, the AUC of both clinical models improved (AUC, 0.91 to 0.93 and 0.88 to 0.91, respectively). The lobe-specific analysis revealed that the nodule-bearing lobes had less emphysema than the rest of the lung regarding benign (EI, 0.5 vs. 0.7 %; *p* < 0.001) and malignant nodules (EI, 1.2 vs. 1.7 %; *p* = 0.001).

**Conclusions:**

Nodules in subjects with higher whole-lung metrics of emphysema and less fibrosis are more likely to be malignant; hereby the nodule-bearing lobes have less emphysema. QCT variables could improve the risk assessment of incidental pulmonary nodules.

**Key Points:**

*• Nodules in subjects with higher whole-lung metrics of emphysema and less fibrosis are more likely to be malignant.*

*• The nodule-bearing lobes have less emphysema compared to the rest of the lung.*

*• QCT variables could improve the risk assessment of incidental pulmonary nodules.*

## Introduction

Increasing use of chest CT, such as for lung cancer screening, and widespread reduction of slice thickness have led to a higher incidence of small pulmonary nodules in clinical routine. Their management remains challenging for clinicians since benign and malignant nodules have ambiguous radiographic features [1]. A previous study suggested that substantial increases in chest imaging and nodule detection produced more false-positive results but at the same time failed to identify more cases of lung cancer [2]. Apart from long-term imaging controls to detect growth, histological work-up, or PET/CT, the probability of lung cancer in incidental pulmonary nodules > 8 mm can be estimated non-invasively by statistical prediction models in clinical routine [3, 4]. Two of the most commonly used models are the Mayo Clinic Model for incidental nodules and the Brock University Model for screening-detected nodules [5, 6]. Both models have been widely validated in several studies based on various populations with both types of nodules [7–11]. However, a recent study based on a large cohort of 23’789 participants with incidental pulmonary nodules reported only an “acceptable” predictive value of both models with a tendency toward lung cancer overestimation [12]. Some groups tried improving risk and outcome prediction by taking the peritumoral environment into account [13–16]. For example, Lee et al reported that combining intratumoral radiomics with peritumoral radiomics improved the predictive value regarding the outcome prediction in NSCLC patients [16]. Therefore, it is tempting to speculate whether a whole-lung approach could add further value to the existing models.

Quantitative computed tomography (QCT) has long been established as an objective method to assess lung parenchymal and airway abnormalities in various diseases such as COPD and interstitial lung disease (ILD) [17–20]. Since COPD and ILD are associated with an increased lung cancer risk, we hypothesized that QCT may yield predictive value concerning malignancy; however, its potential has hardly been explored to date. Thus, the aim of the present study was to explore potential QCT metrics to explain malignancy in incidental pulmonary nodules in 251 subjects in a population at risk using a whole-lung as well as a lobe-based approach. Consecutively, the most promising metrics could be further investigated in larger datasets and potentially be integrated into the Mayo Clinic Model and the Brock University Model.

## Materials and methods

This study was approved by the local ethics committee and conducted in accordance with the principles of the declaration of Helsinki.

### Population

**Fig. 1:**
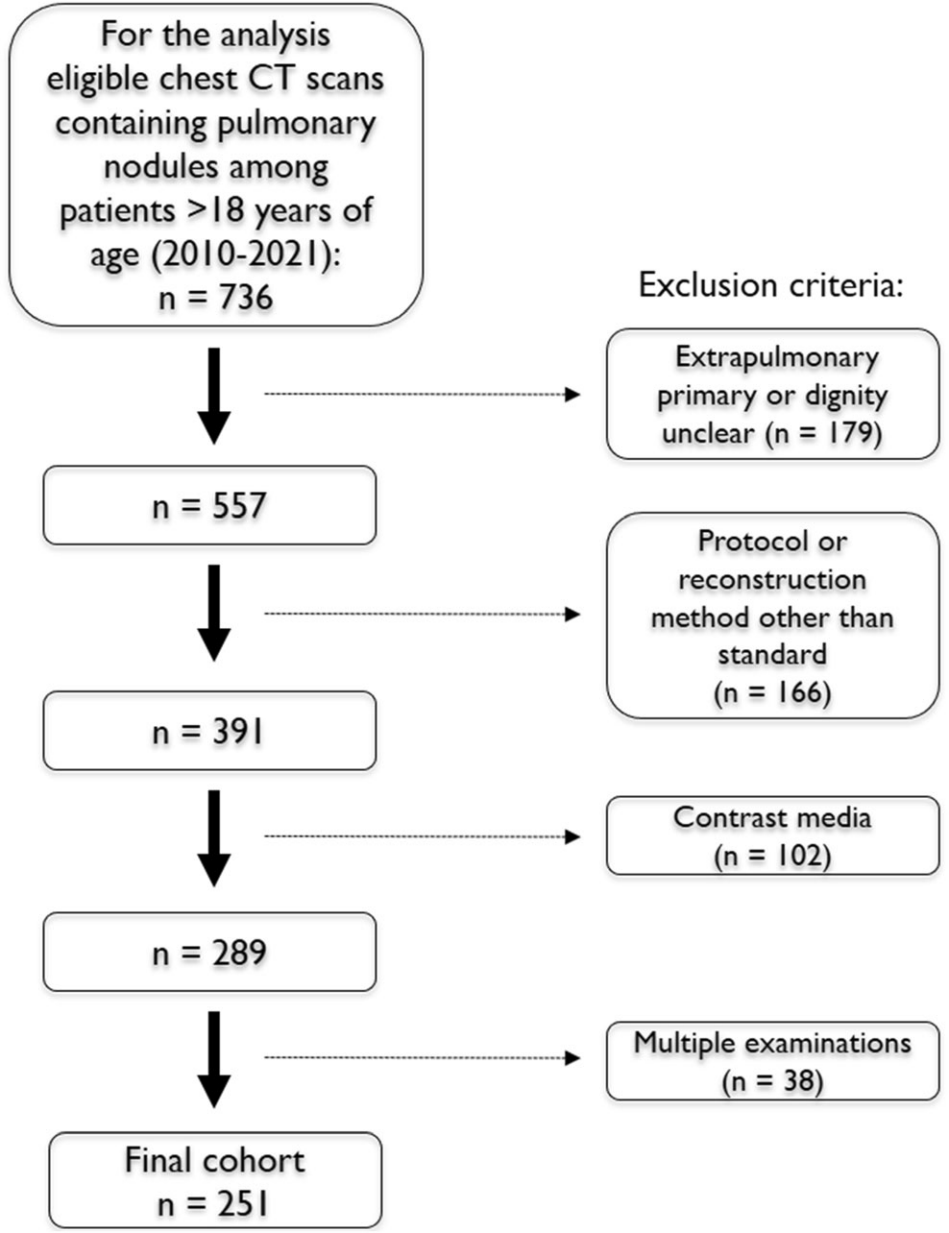
Patient flowchart

For this retrospective exploratory study, patients with chest CT scans between 01/2010 and 12/2021 containing pulmonary nodules were screened. After the strict application of the exclusion criteria, 251 subjects were eligible for the final analysis (Fig. 1). Of note, only one exam per patient was permitted and only one predefined index nodule, the most suspicious or the largest lesion, was analyzed. Clinical information was obtained from the electronic medical records.

### Chest computed tomography parameters

All patients were examined on a 64-row CT scanner (Definition AS64, Siemens, Siemens Medical Solutions) in full inspiration supine position and without intravenous contrast administration. The following acquisition parameters were used: 100–120 kV and 70 mAs reference with dose modulation (Caredose 4D, Siemens), collimation 0.6 mm, reconstructed slice thickness 1.0 mm, and increment 0.8 mm in an iterative medium-soft kernel (I40f, SAFIRE level 3, Siemens).

### Quantitative post-processing

The previously well-evaluated in-house software YACTA (version 2.9.4.16) analyzed the chest CT images fully automated. The airway tree and lung parenchyma were segmented and QCT parameters were calculated for the whole lung as well as individually for each lobe (Table 1) as previously described [21–25]. The overall segmented lung voxels are subdivided into 50% central and 50% peripheral lung zones.

**Table 1:** Quantitative CT (QCT) parameters computed by YACTA

Parameter (unit)	Description
MLD (HU)	Mean lung density
EI (%)	Emphysema index, percentage of emphysema voxel based on threshold −950 HU
pEI (%)	Peripheral emphysema index
EI_CC120_ (%)	Percentage of emphysema contained in emphysema clusters > 120 mm^3^
Perc15 (HU)	15^th^ percentile of lung density histogram
BI (%)	Bulla index
GGOI (%)	Ground glass opacity index, percentage of the segmented lung voxels ≥ −800 HU and < −700 HU
FIBI (%)	Fibrosis index, percentage of the segmented lung voxels ≥ −700 HU
Lung volume (mL)	Volume of segmented lung voxels
WP (%)	Airway wall percentage
AWT-Pi10 (cm)	Airway wall thickness of a theoretical airway with an internal perimeter of 10 mm

### Statistical analysis

All statistical analyses were performed using SPSS Statistics version 25.0. (IBM Corp. 2017) and GraphPad Prism (GraphPad Software, Inc., version 8) under the guidance of a professional statistician. Continuous parameters are reported as median and interquartile range (IQR), if normal distributions are not expected, else mean with standard deviation (SD) is given. Comparisons between the groups were performed using the Mann-Whitney *U* test. Categorical variables are reported as absolute numbers and percentages, and comparisons between the groups were performed using the chi-square test. Additionally to the description, those QCT variables with a descriptive difference (based on median and Mann-Whitney *U* test) were investigated using a univariate logistic regression. Due to the small number of malignant tumor patients (*n* = 51), a backward variable selection based on likelihood ratio tests starting with these QCT variables was then conducted to not endanger the stability of the model. The logistic regression models are evaluated using the areas under the receiver-operating-characteristic curve (AUC) based on all available data. The different models were compared descriptively with a likelihood ratio test. For the nodule-bearing lobe analysis, the parameters were weighted according to their relative share in total lung volume and then compared with the rest of the lung using the Wilcoxon test for paired samples. This is an exploratory analysis. Hence, all *p* values are of descriptive nature and no formal sample size calculation was conducted.

## Results

After the strict application of the eligibility criteria, 251 patients with one defined index lesion each were consecutively included (Fig. 1).

The median [IQR] age of the cohort was 65 [57–73] years, and 37% (*n* = 92) were females. Sixty-three percent were current or former smokers, and the proportion of these ever-smokers was slightly different between the benign and the malignant nodule group (61 vs. 71%).

The malignant nodules all had a histological work-up; the dignity of the benign nodules was proven by either histology (*n* = 11/200) or at least 1-year follow-up (*n* = 189/200). During the follow-up, 13% of the benign nodules had decreased in size or were entirely gone. The mean [SD] nodule diameter was 9.5 [6.9] mm for all patients and differed between the benign vs. the malignant nodule group (7.9 [3.3] vs. 16.0 [11.8] mm) (Table 2). Regarding the applied effective doses, there was no difference regarding the median [IQR] size-specific dose estimation between the benign and the malignant nodule group (5.1 [4.4–5.8] vs. 5.0 [4.1–5.9] mSv).

**Table 2:** Patient demographics and nodule characteristics. Continuous parameters as median (interquartile range), categorical parameters as absolute numbers (percentages)

Demographic/characteristic	Total (*n* = 251)	Benign nodules (*n* = 200)	Malignant nodules (*n* = 51)
Age (years), median (IQR)	65 (57–73)	64 (55–73)	67 (61–76)
Females (*n*, %)	92 (36.7%)	71 (35.5%)	21 (41.2%)
Smoking status (*n*, %)			
Current	29 (11.6%)	22 (11.0%)	7 (13.7%)
Former	128 (51.0%)	99 (49.5%)	29 (56.9%)
Never	39 (15.5%)	30 (15.0%)	9 (17.6%)
Unknown	55 (21.9%)	49 (24.5%)	6 (11.8%)
Lung cancer in family (*n*, %)			
Yes	26 (10.4%)	24 (12.0%)	2 (3.9%)
No	195 (77.7%)	151 (75.5%)	44 (86.3%)
Unknown	30 (12.0%)	25 (12.5%)	5 (9.8%)
COPD (*n*, %)	47 (18.7%)	33 (16.5%)	14 (27.5%)
Emphysema (*n*, %)	62 (24.7%)	44 (22.0%)	18 (35.3%)
ILD (*n*, %)	62 (24.7%)	58 (29.0%)	4 (7.8%)
Rheumatic disease (*n*, %)	13 (5.2%)	13 (6.5%)	0
Pulmonary infection (*n*, %)	8 (3.2%)	8 (4.0%)	0
Nodule diameter (mm)	9.5 (6.9)	7.9 (3.3)	16.0 (11.8)
Nodule attenuation (*n*, %)			
Solid	222 (88.4%)	179 (89.5%)	43 (84.3%)
Part-solid	22 (8.8%)	15 (7.5%)	7 (13.7%)
Ground-glass	7 (2.8%)	6 (3.0%)	1 (2.0%)
Nodule size (*n*, %)			
< 74 mm	10 (4.0%)	10 (5.0%)	0
4–6 mm	45 (17.9%)	43 (21.5%)	2 (3.9%)
> 6–8 mm	77 (30.7%)	75 (37.5%)	2 (3.9%)
> 8–15 mm	94 (37.5%)	66 (33.0%)	28 (54.9%)
> 15 mm	25 (10.0%)	6 (3.0%)	19 (37.3%)
Spiculation (*n*, %)	79 (31.5%)	45 (22.5%)	34 (66.7%)
Nodule localization (*n*, %)			
Upper lobe	95 (37.8%)	75 (37.5%)	20 (39.2%)
Middle lobe/lingula	25 (10.0%)	20 (10.0%)	5 (9.8%)
Lower lobe	131 (52.2%)	105 (52.5%)	26 (51.0%)

*COPD* chronic obstructive pulmonary disease, *ILD* interstitial lung disease, *IQR* interquartile range

### Association of whole-lung QCT and nodule malignancy

**Fig. 2:**
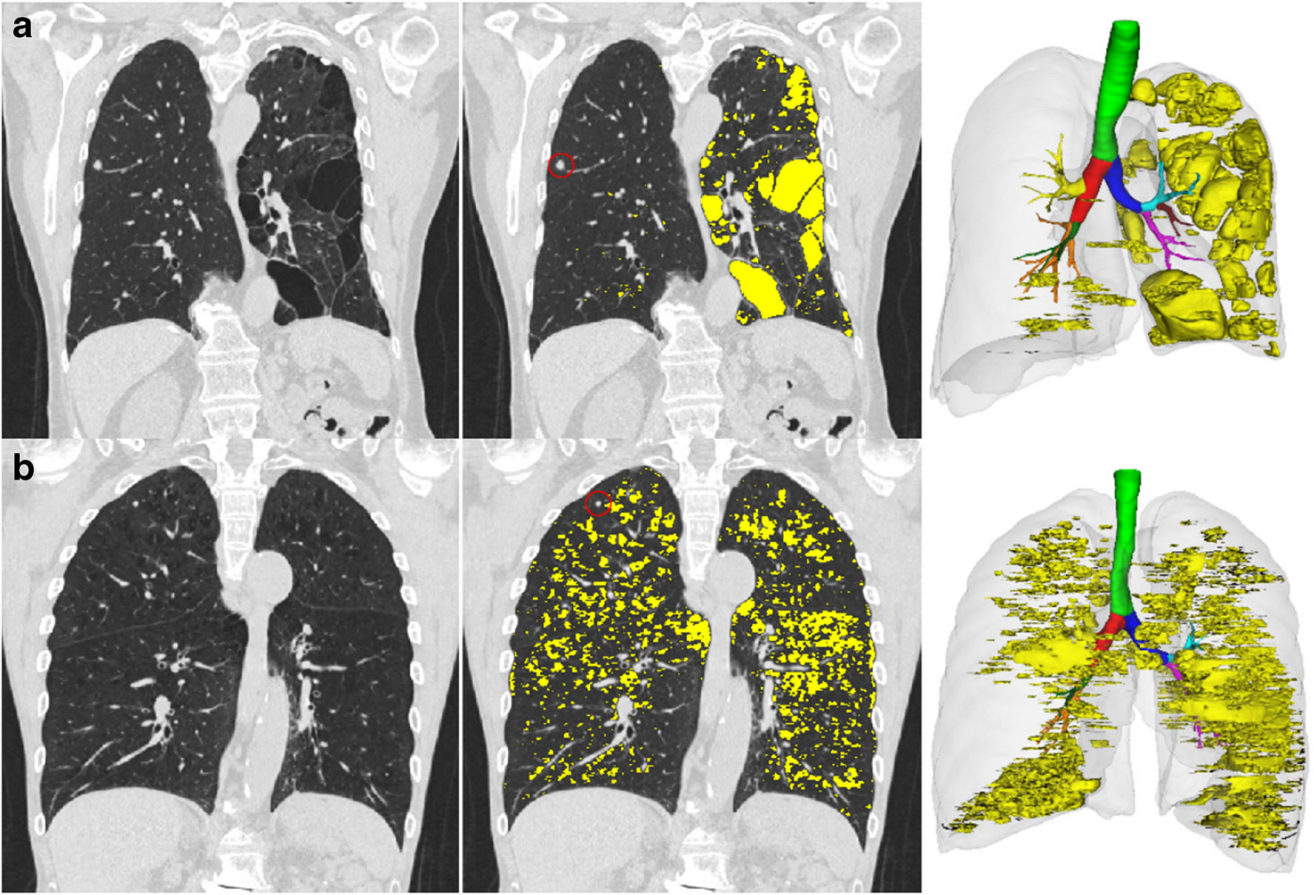
Exemplary YACTA analyses. Top row (**a**): A 75-year-old male with severe, mainly left-sided emphysema (marked in yellow) with an 8 mm nodule in the right upper lobe (red circle). The nodule turned out to be a primary pulmonary adenocarcinoma. The patient had suffered of recurrent pneumothorax caused by this severe one-sided bullous emphysema of unknown etiology since childhood. The 3D volume image on the right depicts the airway segmentation and the large clusters of emphysema in the left lung (whole lung parameters: MLD, −814.7 HU; EI, 10.1%; tumor-bearing lobe parameters: MLD, −824.3 HU; EI, 2.6%). Bottom row (**b**): A 73-year-old male COPD patient (50 py) with a 5 mm nodule in the right upper lobe (red circle). The nodule disappeared during follow-up and therefore was classified as benign. The 3D volume image on the right depicts the airway segmentation and the small clusters of emphysema distributed over both lungs (whole lung parameters: MLD, −827.0 HU; EI, 17.3%; tumor-bearing lobe parameters: MLD, −831.4 HU; EI, 15.6%)

The group comparison showed differences regarding multiple whole-lung QCT parameters (Table 3). Patients with malignant nodules for example had lower MLD, higher EI, and greater lung volumes (Fig. 2). However, they had a lower FIBI and GGOI. There were no differences observed between the groups regarding the airway parameters.

**Table 3:** Quantitative CT parameters (whole lung). Parameters as median [interquartile range]

Parameter	Benign nodules (*n* = 200)	Malignant nodules (*n* = 51)	*p* value^*a*^
MLD (HU)	−766 [−796 to −729]	−789 [−802 to −755]	0.003
EI (%)	0.7 [0.2–2.9]	1.5 [0.4–4.5]	0.030
pEI (%)	40.1 [32.6–51.8]	44.6 [38.5–53.9]	0.026
EI_CC120_ (%)	0.03 [0.00–0.36]	0.19 [0.01–1.33]	0.007
Perc15 (HU)	−907 [−923 to −885]	−915 [−930 to −898]	0.024
BI (%)	0.20 [0.05–1.15]	0.63 [0.09–2.38]	0.015
GGOI (%)	12.5 [7.4–19.6]	8.8 [6.2–17.6]	0.011
FIBI (%)	15.9 [13.4–23.0]	13.9 [12.7– 17.3]	0.008
Lung volume (mL)	5443 [4678–6654]	6233 [4962–7460]	0.025
WP (%)	46.4 [42.9–51.6]	46.2 [42.0–49.4]	0.408
AWT-Pi10 (cm)	0.22 [0.17–0.26]	0.21 [0.16–0.25]	0.787

^a^Mann-Whitney U test

*AWT-Pi10* airway Pi10, *BI* bulla index, *EI* emphysema index, *EI*_*CC120*_ emphysema index cluster class 120, *FIBI* fibrosis index, *GGOI* ground-glass opacity index, *MLD* mean lung density, *pEI* emphysema index of peripheral zone, *Perc15* 15^th^ percentile lung density, *WP* wall percentage

### MLD and pEI are associated with an increased risk of malignancy

Univariate logistic regression analysis showed that multiple parameters are influential variables for pulmonary nodule malignancy. These results are consistent with the description of the QCT variables grouped by malignancy.

MLD, Perc15, GGOI, or FIBI showed a protective association with malignancy (OR < 1). EI, pEI, or BI were associated with an increased malignancy risk (OR > 1). In order to elaborate a multivariate QCT parameter model, a backward variable selection was performed, starting with all parameters with univariate *p* values < 0.05. It resulted in a model consisting of MLD and pEI (Table 4). A ROC-curve based on all available data was constructed for this model, the corresponding AUC was 0.69 (95%-CI, 0.61–0.76).

**Table 4:** Results of the logistic regression analysis.

	Univariate logistic regression			Multivariate logistic regression		
Variable	OR	95%-CI	*p* value	OR	95%-CI	*p* value
MLD	0.988	0.980–0.995	0.002	0.9856	0.9856−0.9934	< 0.001
EI	1.010	0.974–1.048	0.584			
pEI	1.031	1.006–1.056	0.015	1.044	1.015−1.075	0.003
EI_CC120_	1.003	0.956–1.052	0.915			
Perc15	0.989	0.979–0.998	0.02			
BI	1.159	1.014–1.325	0.031			
GGOI	0.950	0.914–0.988	0.01			
FIBI	0.920	0.868–0.979	0.008			
Lung volume	1.111	0.959–1.286	0.160			

*BI* bulla index, *EI* emphysema index, *EI*_*CC120*_ emphysema index cluster class 120, *FIBI* fibrosis index, *GGOI* ground-glass opacity index, *MLD* medium lung density, *OR* odds ratio, *pEI* peripheral emphysema index, *Perc15 15*^*th*^ percentile lung density

### Addition of MLD and pEI potentially improves the Mayo Clinic Model and the Brock Model

**Fig. 3:**
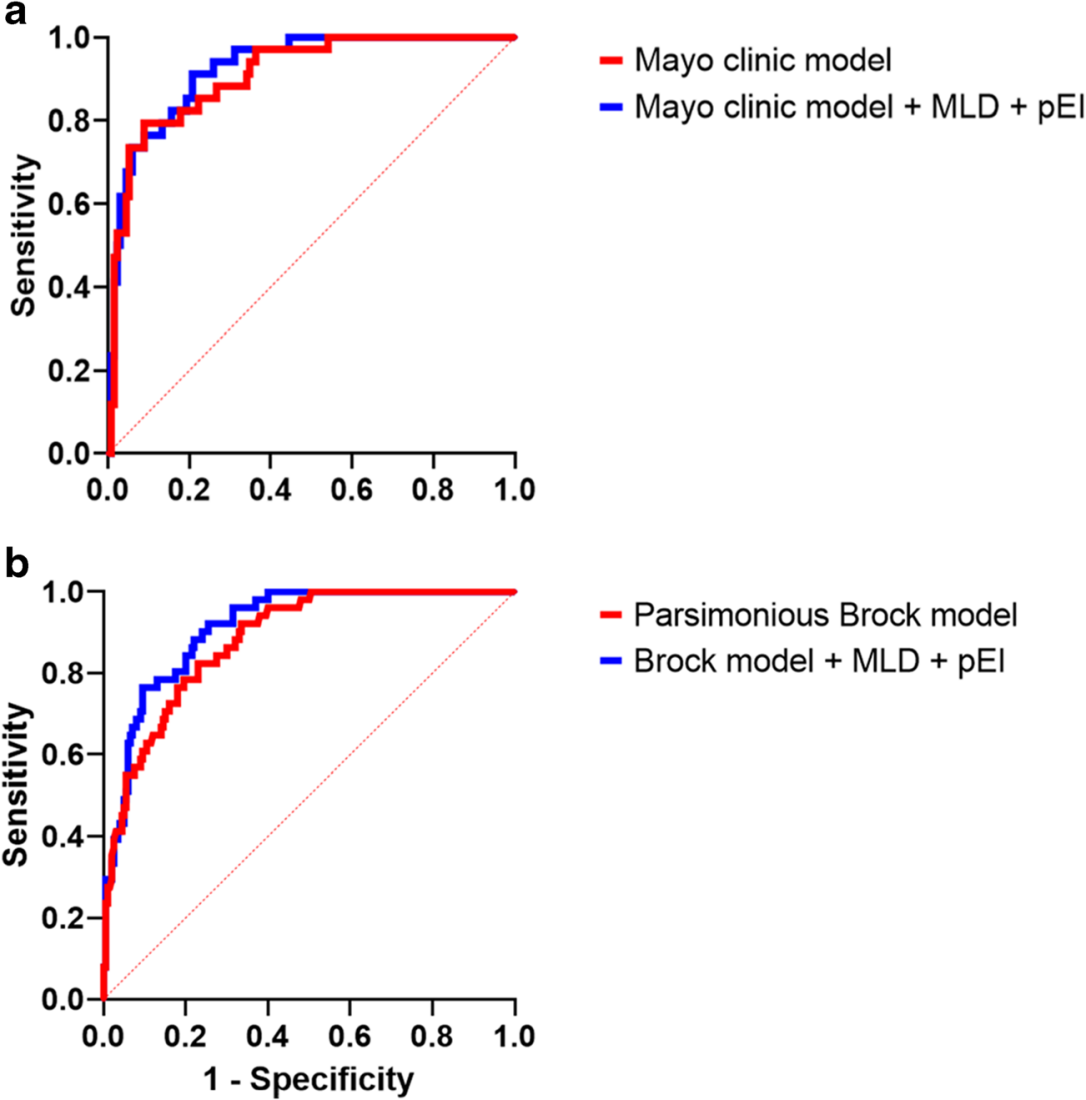
**a** The ROC curves of the Mayo Clinic Model without (red curve) and with (blue curve) the QCT parameters MLD and pEI. **b** The respective ROC curves of the parsimonious Brock Model with (blue curve) and without (red curve) the QCT parameters MLD and pEI

To evaluate a potential benefit of the QCT parameters for the Mayo Clinic Model or the (parsimonious) Brock University Model, MLD and pEI were integrated into these models. In both models, the AUC based on all available data increased slightly after the addition of MLD and pEI: The AUC of the Mayo Clinic Model increased from 0.91 (95%-CI, 0.86–0.96) to 0.93 (95%-CI, 0.88–0.97, *p* = 0.02) and of the parsimonious Brock Model from 0.88 (95%-CI, 0.84–0.93) to 0.91 (95%-CI, 0.88–0.95; *p* < 0.001) (Fig. 3).

### Nodule-bearing lobes show less lung disease than the rest of the lung

To explore the features of the nodule-bearing lobes, a lobe-specific analysis was performed and the QCT parameters of the respective lobes were compared to the rest of the lung per subject. While the MLD was similar, parameters indicating emphysema were lower in the nodule-bearing lobe compared to the rest of the lung similarly in subjects with malignant and benign nodules. In both groups, FIBI and the airway WP were higher in the nodule-bearing lobes compared to the rest of the lung (Table 5).

**Table 5:** Parameters presented as median [interquartile range]

Lobe-specific evaluation						
	Benign nodules (*n* = 200)			Malignant nodules (*n* = 51)		
Parameter	Nodule-bearing lobe	Nodule-free lung	*p* value^*a*^	Nodule-bearing lobe	Nodule-free lung	*p* value^*a*^
MLD (HU)	−760 [−795 to −714]	−767 [−797 to −726]	0.044	−768 [−802 to −737]	−790 [−809 to −759]	0.097
EI (%)	0.5 [0.1–1.9]	0.7 [0.3–3.0]	< 0.001	1.2 [0.3–3.0]	1.7 [0.4–5.1]	0.001
pEI (%)	36.3 [24.6–47.9]	41.9 [31.8–53.9]	< 0.001	38.7 [28.4–46.3]	46.4 [37.9–55.2]	0.012
EI_CC120_ (%)	0.00 [0.00–0.12]	0.03 [0.00–0.41]	< 0.001	0.06 [0.00–0.29]	0.21 [0.01–1.56]	0.002
Perc15 (HU)	−901 [−922–−876]	−908 [−924 to −887]	< 0.001	−909 [−926 to −892]	−918 [−932 to −898]	< 0.001
BI (%)	0.14 [0.02 –0.65]	0.22 [0.06–1.33]	< 0.001	0.41 [0.08–1.11]	0.75 [0.09–2.75]	0.003
GGOI (%)	13.0 [7.2–22.3]	12.1 [7.4–19.7]	0.023	10.1 [6.1–17.8]	8.2 [6.0–16.4]	0.154
FIBI (%)	16.5 [13.0–22.6]	15.7 [13.2–21.8]	0.095	14.5 [12.7–18.5]	13.7 [12.5–17.0]	0.330
WP (%)	50.0 [50.0–56.5]	45.3 [42.1–50.0]	< 0.001	46.6 [37.2–55.0]	44.5 [37.8–47.4]	< 0.001
AWT-Pi10	0.20 [0.16–0.29]	0.20 [0.16–0.26]	0.587	0.18 [0.10–0.24]	0.19 [0.12–0.24]	0.554

^a^Wilcoxon test for paired samples

*AWT-Pi10* airway wall thickness Pi10, *BI* bulla index, *EI* emphysema index, *EI*_*CC120*_ emphysema index cluster class 120, *FIBI* fibrosis index, *GGOI* ground-glass opacity index, *pEI* peripheral emphysema index, *WP* wall percentage

## Discussion

In this retrospective exploratory study, we found that whole-lung QCT metrics may support in identifying malignancy in incidental pulmonary nodules. Furthermore, mean lung density and the emphysema index of the peripheral zone might improve the performance of two established radio-clinical risk models. We additionally observed that in both groups the nodule-bearing lobes had less emphysema, more fibrosis, and more airway wall thickening than the rest of the lung within the same individual. This analysis can serve as a starting point for further research on the explanatory characteristics of QCT variables in pulmonary nodule malignancy.

The fact that lung cancer and emphysema are linked, is well appreciated [26, 27] as they share smoking as an underlying trigger, and our findings agree with the literature, as the whole-lung EI was higher in the malignant compared to the benign nodule group. By using bulla shape-based features, we could furthermore show that the malignant nodule group had a higher proportion of peripheral zone emphysema and larger clusters of emphysema than the benign nodule group.

The whole-lung approach used in this study has several advantages over the widespread tumoral or peritumoral approach: First, it does not have the limitation of region-of-interest (ROI) selection and the connected inter-observer variability. Second, it is highly standardized and therefore not only effort- and time-saving but also enables optimal comparability with follow-up studies. Although the existing literature on lung cancer prediction using whole-lung radiomics is rather sparse, our findings are somewhat consistent with similar studies. For example, Liang et al analyzed a smaller cohort of idiopathic pulmonary fibrosis patients (*n* = 116) and reported that the histogram-based whole-lung radiomics features kurtosis and energy have a significant predictive value regarding lung cancer development, especially if combined with traditional risk factors such as smoking status or age [28]. In the current exploratory study, nine out of thirteen analyzed parameters were different between the benign and the malignant nodule group, and six of them showed a predictive value in the logistic regression analysis. In contrast to Liang et al, the current study not only relied on histogram-based radiomics but also included shape-based radiomics such as the bulla index. As reported by Wiemker et al, these features have the advantage of not only depending on a fixed HU threshold and may also account for irregular shapes and overlapping or open contours of the bullae [29].

One of the relevant advantages of QCT parameters is that they are well-evaluated and generated from routine imaging. Thus, neither additional query, investigation, invasive or otherwise bothering interventions nor costly analyses are necessary.

Regarding the comparison to the standard prediction models, Vachani and colleagues have already pointed out that both, the Mayo Clinic and the Brock University Model, may overestimate cancer probabilities in certain cohorts [12]. In their study, the Mayo Clinic Model had an AUC of 0.75, which was slightly better than the Brock Model, with an AUC of 0.71.

In this study, both models showed an excellent performance; as expected, the Mayo Clinic Model performed slightly better than the Brock Model (AUC, 0.91 vs. 0.88) since it was initially designed for incidental pulmonary nodules.

The derived model from the backward variable selection based on QCT parameters alone already had an AUC of 0.69 on all available data. In consequence, the integration of QCT parameters into the two standard models increased the AUCs to 0.93 and 0.91, respectively. The AUCs were all calculated using the complete dataset, which means that the model is evaluated with the data it was created with. The reason for this unconventional approach is that the amount of data and the number of malignant tumor patients were rather low. Of note, a separation into training, validation, and test sets would have resulted in a small test set with an expected 10 malignant patients (20% of the data) which would not have allowed a robust evaluation of the derived model. The interpretation is therefore strictly descriptive and no definite conclusions about the predictive value of the QCT variables can be drawn. Additionally, it must be noted that the AUCs for the here proposed models are very likely estimated too high, since an evaluation based on the training data results in overfitting.

The lobe-specific analysis of QCT parameters revealed that amongst others, the nodule-bearing lobes had lower EI, lower EI in the peripheral zone, and smaller BI compared with the rest of the lungs. Interestingly, the benign and the malignant nodule groups did not show any differences in the lobar approach. One possible explanation for this finding is that there is less functional lung parenchyma and less blood supply in areas of emphysema and that thus neoplasms of any kind may be less probable in lobes with fewer grounds to grow on. Literature on the relationship between emphysema and lung cancer remains controversial. For example, Hohberger et al stated that higher regional emphysema scores are associated with the presence of lung cancer in a cohort containing 624 malignant nodules using a semiquantitative approach [30]. In contrast to the current study, this approach is based on a subjective estimate rather than objectively measured values. Other studies using a quantitative approach failed to show a link between emphysema and lung cancer at all [31, 32]. In the lobe-specific analysis, we observed that the WP was higher in the nodule-bearing lobes compared with the rest of the lungs. Similar to the emphysema-associated parameters, this accounted for both, the benign and the malignant nodule group and is most probably based on the underlying inflammatory or malignant process. However, this finding needs to be elaborated in future studies, e.g. to evaluate the predictive value of different types of bronchial wall thickening regarding malignancy.

This study has several limitations. The most relevant one is the lack of external validation regarding the proposed QCT- and the expanded models because they were so far only evaluated on the same data used to derive the models. Therefore, further validation of the models on an independent dataset is warranted. Furthermore, the algorithm could not segment and therefore not exclude the nodules from the analysis, which might have affected the QCT parameters.

However, this effect would only account for the density-based parameters MLD, EI, and FIBI. It can be assumed that the current results of the whole lung analysis would have been even more significant after the exclusion of the nodules, since the malignant nodules were larger and their voxels contribute to the higher HU values in the lungs. This leads, for example, to the fact that the differences for MLD, FIBI, and EI between groups would become even larger. Regarding the lobar approach, the relative contribution of a nodule to the lobe-based CT parameters could be greater than in the whole-lung approach. Thus, an exclusion of the nodules indeed might affect the density-based QCT parameters (MLD, EI, and FIBI) of the respective lobe and could lead to a lower MLD, higher EI, or lower FIBI in the respective lobe. However, the other emphysema-related parameters (pEI, EI_cc120_, Perc15, and BI) can be assumed to be robust to this effect, since they rather describe the distribution and clustering of the emphysema instead of being solely based on the voxel density.

Then, the recruitment occurred in a dedicated chest hospital with frequent referrals of patients with severe ILD and COPD, presenting with incidental nodules. Thus, our results may not be readily transferable to screening populations. The sample size was relatively small and only contained 51 malignant nodules. However, our cohort is clinically well characterized and CT protocols were strictly standardized, which was necessary to ensure comparability regarding the QCT parameters [33, 34]. This led to the exclusion of a great number of cases. Lastly, depending on the CT scanner and its specific settings, the quantitative results may vary, however, they should be consistent within the same institution. Future endeavors should focus on the harmonization of imaging protocols facilitating the advance of QCT analysis and fostering cross-institutional standardization and reproducibility.

In conclusion, the present study demonstrates that QCT parameters of the whole lung may be considered for malignancy risk assessment in incidental pulmonary nodules. QCT might add value in combination with the established Mayo Clinic and Brock University Model.

## Abbreviations

AUC: Area-under-the-curve
AWT-Pi10: Airway wall thickness (theoretical airway with an internal perimeter of 10 mm)
BI: Bulla Index
COPD: Chronic obstructive pulmonary disease
CT: Computed tomography
EI: Emphysema index
FIBI: Fibrosis index
GGOI: Ground-glass opacity index
ILD: Interstitial lung disease
IQR: Interquartile Range
MLD: Mean lung density
pEI: Peripheral emphysema index
Perc15: 15^th^ percentile lung density
QCT: Quantitative computed tomography
ROC: Receiver operating characteristic
WP: Wall percentage

## Declarations

### Guarantor

The scientific guarantor of this publication is Dr. Peters.

### Statistics and biometry

One of the authors has significant statistical expertise (Dr. Moritz Pohl, MSc; Institute of Medical Biometry, University of Heidelberg, Heidelberg, Germany).

### Methodology

• diagnostic or prognostic study

• performed at one institution
